# Cardiovascular Disease and miRNAs: Possible Oxidative Stress-Regulating Roles of miRNAs

**DOI:** 10.3390/antiox13060656

**Published:** 2024-05-27

**Authors:** Seahyoung Lee

**Affiliations:** Department of Convergence Science, College of Medicine, Catholic Kwandong University, Gangneung-si 25601, Republic of Korea; sam1017@ish.ac.kr

**Keywords:** microRNA, oxidative stress, cardiovascular system

## Abstract

MicroRNAs (miRNAs) have been highlighted as key players in numerous diseases, and accumulating evidence indicates that pathological expressions of miRNAs contribute to both the development and progression of cardiovascular diseases (CVD), as well. Another important factor affecting the development and progression of CVD is reactive oxygen species (ROS), as well as the oxidative stress they may impose on the cells. Considering miRNAs are involved in virtually every biological process, it is not unreasonable to assume that miRNAs also play critical roles in the regulation of oxidative stress. This narrative review aims to provide mechanistic insights on possible oxidative stress-regulating roles of miRNAs in cardiovascular diseases based on differentially expressed miRNAs reported in various cardiovascular diseases and their empirically validated targets that have been implicated in the regulation of oxidative stress.

## 1. Introduction

Cardiovascular diseases (CVDs) have been the leading cause of death worldwide for many decades, and the number of deaths from CVDs such as ischemic heart disease, ischemic stroke, and hypertensive heart disease was estimated to be 14.72 million in 2021 [1]. As multifactorial disorders, various risk factors have been identified for CVDs, including but not limited to hypertension, high blood cholesterol levels, stress, smoking, diabetes, and obesity [2]. At the molecular level, the majority of those risk factors can be, at least partially, linked to the excessive production of reactive oxygen species (ROS) [3]. Considering that ROS act as important second messenger signaling molecules facilitating post-translational protein modifications in many different aspects of cell biology [4], it is not surprising to know that excessive ROS has been associated with various CVDs such as heart failure, hypertension, and atherosclerosis [5,6,7]. 

Similarly, microRNAs (miRNAs) play a significant role in a wide range of biological processes, not to mention the development and progression of human diseases. More than 60% of mammalian messenger RNAs (mRNAs) are expected to be regulated by miRNAs, and a single mRNA can be targeted by multiple miRNAs [8]. This unique mutually multi-targeting feature of miRNAs insinuates that they are important cellular regulators to fine-tune the expression of hundreds of proteins [9], and accordingly, miRNAs have also been implicated in the development and progression of various CVDs [10,11]. 

Now, it is a well-accepted consensus that ROS and miRNAs mutually regulate each other during the development and progression of CVDs, and many comprehensive reviews on the subject are available [12,13,14]. Nevertheless, due to the complexity of both ROS- and miRNA-mediated regulation of biological processes, it is reasonable to think that a lot more ROS–miRNA interactions need to be unraveled. Therefore, conducting meticulously designed experiments is important to discover unknown mechanisms regarding the subject, but it may also be important to generate and examine hypothetically possible scenarios in advance so that researchers can expand the scope of research and better interpret rather seemingly contradicting results they may face in their research. 

To meet such demands, and also to differentiate from other reviews, this narrative review will take an approach different from conventional ones. For one, the major purpose of this narrative review will be to provide mechanistic insights on the possible oxidative stress regulating roles of miRNAs in CVDs rather than simply summarize known facts or previously published results regarding the subject. 

To serve the purpose, first, a brief introduction to the basics of ROS and miRNAs will be given, keeping it to a minimum. For the rest of the review, theoretically possible regulatory interactions between ROS and miRNAs will be formulated based on the miRNAs reported to be differentially expressed in various CVDs and their empirically validated targets known to be involved in the regulation of oxidative stress. Also, their possible impact on the cardiovascular system will be briefly discussed. 

## 2. ROS at Glance

ROS is a term that collectively refers to a group of highly reactive, oxygen-containing molecules generated by redox reactions or by electronic excitation. They include oxygen free radicals, such as superoxide anion radicals (O_2_·−), hydroxyl radicals (·OH), and peroxyl radicals (HO_2_·), as well as non-radicals, such as hydrogen peroxide (H_2_O_2_), hypochlorous acid (HOCl), and ozone (O_3_) [15]. The generation of ROS is mainly facilitated by the mitochondrial electron transport chain and a number of redox-catalyzing enzymes such as nicotinamide adenine dinucleotide phosphate (NADPH) oxidases, xanthine oxidases (XO), nitric oxide synthases (NOS), cyclooxygenases (COX), cytochrome P450 enzymes (CYP), and lipoxygenases (LOX) [16]. 

Additionally, it is known that various environmental factors, such as exposure to ultraviolet rays (UV), cigarette smoking, or excessive alcohol consumption, can increase ROS production [17]. The generation of ROS is an unavoidable aspect of aerobic life, and physiologic levels of ROS are required for various cellular functions, such as signal transduction, defense against foreign organisms, and gene expression [18]. However, ROS can be beneficial or deleterious depending on their intracellular level [19]. Therefore, cells are equipped with antioxidant systems to counteract the excessive production of ROS. 

The antioxidant system is comprised of both antioxidant enzymes and non-enzymatic antioxidants [20]. Possibly, the most well-known example of antioxidant enzymes a superoxide dismutases (SODs). SODs catalyze the transformation of superoxide anion radicals (O2·–) into hydrogen peroxide (H_2_O_2_), and by doing so, SODs inhibit the interaction between superoxide anion radicals (O_2_·−) and nitric oxide (NO), preventing the generation of peroxynitrite (ONOO-) [21], an endogenous toxicant [22]. Additional antioxidant enzymes such as catalase (CAT), glutathione peroxidase (GPx), glutathione-S-transferase (GST), and glucose-6-phosphatedehydrogenase (G6PD) also work together to maintain the physiologic level of ROS. 

As for non-enzymatic antioxidants, any proteins having a thiol group (-SH) can function as antioxidants because thiols, as electron acceptors, can reduce unstable free radicals by oxidizing them. Glutathione (GSH) is one of the major intracellular thiols in the cells, while albumin is the plasma equivalent of GSH. Additionally, vitamins C and E function as antioxidants as well [23]. Since the main focus of this review is on the interaction between ROS and miRNAs in relation to the development and progression of CVDs, the detailed mechanisms of the production and the role of ROS and antioxidants are kept to a minimum for this particular review. For more comprehensive, in-depth information on these subjects, the following reviews are recommended [15,16,19]. 

As briefly mentioned above, the physiologic level of ROS within the detoxification capacity of the cells plays an important role in cellular signaling and function by fine-tuning gene expression, cell growth, migration, differentiation, and even death [16,24]. Nevertheless, when ROS production exceeds the antioxidant capacities, so-called oxidative stress occurs, and chronic, accumulated oxidative stress can lead to a wide variety of diseases such as cancer [25], neurodegenerative disease [26], respiratory disease [27], and kidney disease [28]. Furthermore, particularly important for this review, oxidative stress has been associated with a range of CVDs, such as atherosclerosis, ischemic heart disease, hypertension, cardiomyopathy, cardiac hypertrophy, and congestive heart failure [29,30,31]. In addition, their contribution to the development of vascular disease has also been well-reported, and the following reviews will provide good information on the subject [32,33,34].

## 3. MicroRNAs at Glance

Approximately 20-nucleotide-long, non-coding miRNAs bind to their respective target mRNAs for their degradation and/or translational repression [35]. MiRNAs are initially synthesized as thousands of nucleotides long double-stranded primary miRNAs (pri-miRNAs) by the RNA polymerase II in the nucleus. Then, pri-miRNAs are processed by the ribonuclease II Drosha, generating approximately hundreds of nucleotides long miRNAs with a hairpin-liked structure called premature miRNAs (pre-miRNAs). The export of pre-miRNAs from the nucleus to the cytosol is facilitated by the nuclear export factor Exportin 5, and the exported pre-miRNAs are further processed by the ribonuclease III Dicer to produce double-stranded mature miRNAs [36]. The mature miRNAs promptly separate into two strands, and the more stable strand binds to argonaute (Ago) protein to form an RNA-induced silencing complex (RISC) [37]. In the RISC, miRNA functions as a template to recognize the complementary sequence in the 3′ untranslated region (UTR) of target mRNAs. Once the target mRNA complexes with the RISC, miRNA either hinders the target mRNA translation or degrades the target mRNA to achieve the gene silencing of the target mRNA [38]. For the last few decades, miRNAs have been spotlighted as key regulators of virtually every cellular process [39]. Especially, their importance in fine-tuning protein expression has been highlighted, and more than 60% of mammalian mRNAs are expected to be regulated by miRNAs [8]. Since their first discovery in *C. elegans* in 1993 [40], miRNAs have been implicated in various diseases, including CVDs [41,42,43].

## 4. Differentially Expressed miRNAs in CVDs and Oxidative Stress

**Figure 1:**
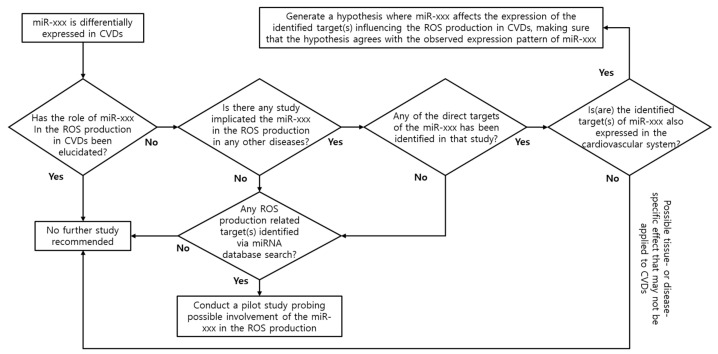
Example of developing a study topic from differentially expressed miRNA data in a certain disease regarding a specific pathological phenotype (for better understanding, CVDs and ROS production are used here as examples).

To provide mechanistic insights on the possible roles of miRNAs in the regulation of oxidative stress regarding CVDs, a theoretically possible regulatory interaction between ROS and miRNAs is hypothesized and discussed for the rest of the review by linking seemingly distant and scattered information on the subject following logic similar to that depicted in Figure 1.

To select miRNAs to be covered in this review, first, the miRNAs whose altered expression has been reported in CVDs were searched from currently available reviews [10,14,43,44]. One by one, the miRNAs from those reviews were further checked for the verification of their differential expression in CVDs by backtracking the original publication. Also, individual miRNA was checked for a more recent update regarding its role in CVDs via PubMed search. Furthermore, each miRNA was also checked for any involvement in the regulation of oxidative stress and/or ROS production based on currently available literature through a PubMed search using keyword combinations such as ‘miR-xxx and oxidative stress’ or ‘miR-xxx and ROS’.

The resultant papers were carefully reviewed, especially focusing on whether the direct target(s) of a given miRNA was empirically validated or not. In this process, studies conducted in non-cardiovascular systems were also included, as long as the expression of the identified target(s) of the corresponding miRNA was verified in the cardiovascular system. In the end, the miRNAs whose differential expression in CVDs was verified, and the involvement in the regulation of oxidative stress with empirically verified targets were subjected to this review. 

For organizational purposes, miRNAs were categorized as ‘up-regulated’, ‘down-regulated’, or ‘varied’. One of the important criteria for the categorization was the manifestation in human samples, if available, and the manifestation in animal studies followed. Additionally, studies reporting different expressions of miRNAs in the blood or serum were not used for the categorization because the expression pattern of tissue miRNAs and that of circulating miRNAs do not necessarily correlate [45,46,47], and more importantly, it is extremely difficult even to speculate on the effect of circulating miRNAs on certain specific organs, tissues, or cells (i.e., heart). Nevertheless, those studies were mentioned in the discussion of the corresponding miRNAs, if necessary. 

### 4.1. Up-regulated miRNAs in CVDs and Their Possible Roles in the Oxidative Stress Regulation

#### 4.1.1. miR-15

It has been reported that the expression of miR-15 was significantly increased in the infarcted area of both porcine and mice cardiac tissue following an ischemia/reperfusion (I/R) injury, and the suppression of miR-15 reduced the infarct size, enhancing the cardiac function [48]. In another animal model of Dicer depletion-induced cardiac dysfunction, the expression of miR-15b was also observed to be increased, and the anti-miRNA-based suppression of miR-15b restored the heart function and attenuated hypertrophy [49]. Regarding the role of miR-15b in the regulation of oxidative stress, it was demonstrated that miR-15b is involved in mitochondrial ROS production by directly targeting sirtuin 4 (SIRT4) [50]. 

SIRTs (SIRTs 1–7) are a family of nicotinamide adenine dinucleotide (NAD)-dependent histone deacetylases that can deacetylate histone and non-histone targets. Furthermore, they have been linked to several antioxidant and oxidative stress-related processes [51]. Among these SIRTs, SIRT4 is somewhat unique because it can induce ROS production, but it can also exert anti-oxidative effects, as well. For example, overexpression of SIRT4 increased ROS production both in the heart and mitochondria in a mouse model of angiotensin II (Ang II)-induced cardiac hypertrophy [52]. Furthermore, in the same study, it was demonstrated that SIRT4 inhibited the binding of superoxide dismutase 2 (SOD2) to SIRT3, thereby reducing the antioxidant activity of SOD2. On the other hand, the knockdown of SIRT4 in primary mouse hepatocytes increased fatty acid oxidation and oxygen consumption [53]. Considering that fatty acid oxidation is a key source of mitochondrial ROS [54], it may be possible that SIRT4 also plays anti-oxidative roles by regulating fatty acid oxidation depending on the cell types. However, at least in the heart, the suppression of miR-15 improved the overall cardiac function, and that may indicate that down-regulation of SIRT4 is more beneficial because SIRT4 plays pro-oxidative roles in the heart or cardiac cells under pathologic conditions. Nevertheless, further studies on the role of miR-15-mediated SIRT4 regulation in terms of oxidative stress in CVDs are necessary to prove or disprove such speculation. 

#### 4.1.2. miR-17-92 Cluster

In humans, the miR-17-92 cluster is located on chromosome 13 open reading frame 25 (C13orf25), and it comprises six miRNAs, namely, miR-17, miR-18a, miR-19a, miR-20a, miR-19b-1, and miR-92a-1 [55]. It has been reported that the expression of the members of the miR-17-92 cluster was increased in chronic hypoxia-induced pulmonary artery hypertension (PAH) [56]. Also, the overexpression of miR-17-92 in cardiac and smooth muscle tissues induced hypertrophic cardiomyopathy and arrhythmia by directly repressing the expression of phosphatase and tensin homolog deleted on chromosome 10 (PTEN) and connexin 43 (Cx43) [57] in animals. In addition, a more clinically relevant study that analyzed the dysregulated miRNAs from the cardiac tissue of hypertrophic cardiomyopathy (HCM) patients identified the increase in miR-17-5p as one of the biomarkers for HCM [58].

Evidence that the miR-17-92 cluster participates in the regulation of ROS was demonstrated in a previous study that investigated the role of miR-92a in a diabetic db/db mice model [59]. In this particular study, heme oxygenase 1 (HO-1) was identified as one of the targets of miR-92a. Since HO-1 is a key enzyme that degrades heme to produce biliverdin and bilirubin, and both of them can serve as antioxidants by scavenging or neutralizing ROS [60], it seems to make sense that the inhibition of miR-92a preserved endothelial function by maintaining HO-1 production, suppressing oxidative stress [59]. Interestingly, it also has been reported that ROS can induce the transcription of the miR-17-92 cluster by stimulating transcription factors such as c-myc, nuclear factor kappa-light-chain-enhancer of activated B cells (NF-κB), and p53 [61]. Therefore, the ROS and miR-17-92 may form a positive feedback loop under pathologic conditions to enhance ROS production. 

#### 4.1.3. miR-21

It has been reported that the expression of miR-21 was significantly up-regulated in right ventricle samples from patients with arrhythmogenic right ventricular cardiomyopathy (ARVC) [62]. Furthermore, another study examined the differential expression of miRNAs in cardiomyopathy and identified miR-21 as one of the significantly up-regulated miRNAs in both hypertrophic (HCM) and dilated cardiomyopathy (DCM) [63]. The endogenous expression level of miR-21 is known to be high in cardiovascular-related cells, including cardiomyocytes (CMs), vascular endothelial cells (VECs), cardiac fibroblasts (cFBs), and vascular smooth muscle cells (VSMCs) [64]. Naturally, its role in myocardial I/R injury has been extensively studied. 

Regarding myocardial I/R injury, miR-21 suppressed myocardial apoptosis in rats [65] and inhibited left ventricular remodeling in the early phase of I/R injury [66]. In addition, restoring the expression of miR-21 that was decreased by myocardial I/R injury was shown to be effective in attenuating the myocardial I/R injury [67]. The most frequently suggested mechanism by which miR-21 exerts protective effects during I/R injury is enhanced anti-apoptotic signaling by targeting PTEN, a well-known pro-apoptotic gene [68]. However, the role of miR-21 on ROS production or oxidative stress regulation in CVDs mostly remains elusive. 

Currently, there are only a few studies available that examine the relationship between miR-21 and oxidative stress (or ROS production) in CVDs. One of them demonstrated that miR-21 mediated the cardio-protective effect of salidroside, a phenylpropanoid glycoside, in CMs exposed to hypoxia/reoxygenation (H/R) treatment [69]. According to this particular study, salidroside suppressed H/R-induced ROS generation while increasing the activities of SOD and GPx in CMs. However, inhibition of miR-21 using anti-miR-21 abrogated all of such beneficial effects of salidroside suggesting that miR-21 facilitated the salidroside-induced down-regulation of ROS production. Nevertheless, that particular study did not elucidate detailed underlying mechanisms of how miR-21 achieved the down-regulation of ROS. On the other hand, although they were not conducted in the context of CVDs, studies have demonstrated miR-21-induced ROS production with detailed mechanisms. 

For example, in a study that examined the relationship between miR-21 and ROS production in ECs exposed to high or oscillating glucose, it was demonstrated that miR-21 promoted the accumulation of cellular superoxide in response to glucose stimulation by directly targeting the ROS-homeostatic genes, namely Krev/Rap1 interaction trapped-1 (KRIT1) and SOD2 [70]. Considering that down-regulation of KRIT1 was reported to be strongly correlated to both increased ROS and decreased SOD2 expression [71], the suggested mechanism of action seems to be legitimate in the given experimental settings. Also, another study examined the role of miR-21 in human epithelial cells exposed to ionizing radiation (IR) and reported similar results. To be more specific, the delivery of exogenous miR-21 to the cells increased the cellular ROS level, and it further enhanced an IR-induced increase in ROS [72]. As for the underlying mechanism, it was shown that miR-21 directly targeted both SOD3 and tumor necrosis factor α (TNFα), which are known to stimulate transcription of SOD2 [73]. 

As such, there is contradicting evidence on the role of miR-21 in the regulation of ROS, and therefore, it is imprudent to decisively state whether miR-21 has beneficial impacts on the cardiovascular system in terms of the regulation of oxidative stress. Further studies focusing on the role of miR-21 in the regulation of ROS or oxidative stress under cardiovascular-related pathologic conditions are recommended. 

#### 4.1.4. miR-22 

MiR-22 is one of the striated muscle tissue (cardiac and skeletal muscles) enriched miRNAs, and it is the most abundant miRNA in the heart [74]. Increased expression of miR-22 has been described in various CVD models. For example, increased miR-22 expression has been reported in cardiac hypertrophy models, either induced by transverse aortic constriction (TAC) or isoproterenol [75,76]. Furthermore, various hypertrophic stimuli such as phenylephrine and Ang II also induced the expression of miR-22 in vitro [75,77]. Experimentally verified targets of miR-22 in the heart or CMs include SIRT1 and peroxisome proliferator-activated receptor-gamma coactivator 1alpha (PGC1-α) [78], and they may participate in miR-22-mediated regulation of oxidative stress. 

PGC-1α is known to be involved in fatty acid oxidation and mitochondrial biogenesis, and down-regulation of PGC-1α can increase ROS production [79]. This inverse correlation between PGC-1α and ROS production seems to stem from its role as a transcriptional regulator of the mitochondrial antioxidant defense system. In a previous study on the role of PGC-1α in the regulation of antioxidant gene expression in ECs, it was shown that PGC-1α increased the expression of mitochondrial antioxidant proteins, such as manganese superoxide dismutase (MnSOD), peroxirredoxins 3 and 5 (Prx3 and 5), mitochondrial thioredoxin (TRX2), mitochondrial thioredoxin reductase (TRXR2), and CAT [80], demonstrating its significance as a key regulator of the mitochondrial ROS detoxification system. Additionally, the transcriptional activity of PGC-1α is affected by multiple post-transcriptional modifications, including acetylation [81], and the NAD+-dependent protein deacetylase SIRT1 can deacetylate PGC-1α [82]. In other words, SIRT1 can enhance the transcriptional activity of PGC-1α by deacetylating it. 

Interestingly, unlike the well-known inhibitory role of miRNA, miR-22 increased the expression of SIRT1 by directly binding to the 3’ untranslated region (UTR) of SIRT1 mRNA [83]. Although miRNA-mediated enhancement of translation is rare, it does happen, and the underlying mechanisms are yet to be elucidated [84]. Therefore, theoretically, miR-22 can promote SIRT1 expression and/or suppress PGC-1α. The only problem is the up-regulation of SIRT1 and down-regulation of PGC-1α have seemingly contradictory effects in terms of oxidative stress. Nevertheless, overexpression of miR-22 did attenuate oxidative stress-induced injury in diabetic cardiomyopathy [83], which may be because the deacetylation of already existing PGC-1α by SIRT1 has much more impact than the miR-22-mediated suppression of PGC-1α translation in terms of the oxidative stress regulation. 

#### 4.1.5. miR-23

In a mouse model of myocardial infarction (MI), miR-23 expression was reported to be up-regulated, causing CM apoptosis [85]. Furthermore, myocardial I/R injury increased the miR-23 expression in rats [86]. Regarding the role of miR-23 in the regulation of oxidative stress, a previous study examining the role of miR-23 in an intestinal I/R injury model may provide some useful insight to predict its role in the cardiovascular system [87]. According to that particular study, the intestinal I/R injury in mice significantly increased the expression of miR-23, and peroxisome proliferator-activated receptor alpha (PPARα) was a direct target of miR-23 [72]. 

PPARα is expressed in various cardiovascular cells, and its cardio-protective effect against myocardial I/R injury has also been reported [88,89]. Most early studies utilized PPARα-selective ligands. For example, a selective PPARα agonist clofibrate suppressed ROS production and lipid peroxidation in a rat model of coronary artery occlusion-induced MI, and such beneficial effects were mainly due to the significantly increased expression of antioxidant genes such as SOD1, SOD2, and CAT [89]. In another study using Wy14643, another synthetic ligand of PPARα, it was also demonstrated that the activation of PPARα protected rabbit hearts from I/R injury by up-regulating HO-1 expression [90]. 

In line with these previous reports, the aforementioned study also demonstrated that either the suppression of miR-23 or the overexpression of PPARα resulted in an increase in antioxidant genes, namely, forkhead box O3 (FOXO3a), PGC-1α, nuclear factor erythroid 2–related factor 2 (Nrf2), CAT, NAD(P)H: quinine oxidoreductase-1 (NQO1), HO-1, and SOD2 [72], suggesting that miR-23 may have a significant impact on the regulation of oxidative stress. 

#### 4.1.6. miR-27 

It has been reported that the endogenous expression of miR-27a was significantly increased following TAC surgery in mice [91]. Also, MI-induced expression of miR-27a in a rat model of chronic heart failure (CHF) has been reported [92]. In that particular study, it was demonstrated that miR-27a directly targeted Nrf2, which is known to regulate the expression of a range of antioxidant enzyme genes such as GST, SODs, HO-1, and NQO1 [93].

Under non-stressed conditions, Nrf2 is bound to its negative regulator Kelch-like ECH-associated protein (Keap1), which facilitates the ubiquitination and subsequent degradation of Nrf2 by E3 ubiquitin ligase Cullin 3 (Cul 3) [94,95]. Consequently, low basal expression of Nrf2 is maintained under non-stressed conditions. However, oxidative stress causes modification of the stress-sensing cysteine residue of Keap1, and that prevents the Keap1-mediated ubiquitination of Nrf2 by Cul3, leading to the accumulation and translocation of Nrf2. The nuclear translocated Nrf2 heterodimerizes with small Maf (sMaf), and this Nrf2-sMaf complex binds to the antioxidant response element (ARE) and initiates robust expression of the antioxidant genes for the detoxification of oxidants such as ROS [96]. Therefore, down-regulation of Nrf2 by miR-27a in CVDs such as MI may contribute to the development and progression of disease by disrupting oxidative stress defensive mechanisms. 

#### 4.1.7. miR-28

In the above-mentioned study, which demonstrated an MI-induced increase in miR-27a, miR-28 was also identified as one of the MI-induced miRNAs [92]. Furthermore, miR-28 was also demonstrated to regulate the Nrf2 expression by directly binding to the 3’UTR of Nrf2 mRNA [97]. Therefore, miR-28 is another example of miRNAs that may have a negative impact on the cardiovascular system by down-regulating Nrf2 in a Keap1-independent mechanism in terms of oxidative stress.

#### 4.1.8. miR-34a

Mir-34a is another miRNA demonstrated to be up-regulated by MI in animal models [98,99]. Regarding its involvement in oxidative stress regulation, it was reported that the suppression of miR-34a significantly increased the expression of antioxidant genes, such as Nrf-2, NQO1, GST, and HO-1, in a rat model of hepatic I/R injury. However, direct targeting of those genes by miR-34a was not empirically demonstrated [100]. Interestingly, it was also reported that SIRT1, one of the antioxidant regulators [101], was one of the direct targets of miR-34a [102].

One of the mechanisms with which SIRT1 exerts antioxidant response is modulating key redox-related transcription factors, such as FOXO3a. The FOXO family of transcription factors has been reported to regulate the expression of a wide variety of genes participating in the antioxidant defense system [103]. For example, antioxidant enzymes such as MnSOD and CAT have FOXO3a binding sites in their promoters, so the activation of FOXO3a induces the transcription of these antioxidant enzymes [104]. This antioxidant effect of FOXO3a was demonstrated in cardiac microvascular endothelial cells (cMVECs), as well [105]. The transcriptional activity of FOXO3a is known to be dependent on its post-translational modifications, such as deacetylation or phosphorylation [106], and SIRT1 is known to deacetylate FOXO3a, inducing antioxidant responses [107]. Therefore, it is reasonable to speculate that the down-regulation of SIRT1 by miR-34a will have a negative impact in terms of oxidative stress, and in fact, such miR-34a-induced oxidative stress has been demonstrated in pathologic conditions, including MI [98,108]. 

#### 4.1.9. miR-93 

The expression of miR-93 has been reported to be up-regulated in animal models of MI [109], carotid artery balloon injury [110], and hindlimb ischemia [111]. Although another study using an animal model of I/R injury reported down-regulation of miR-93 [112], the discrepancy may be due to the different sampling time point since the study reporting down-regulation took the heart sample immediately after the I/R injury as opposed to 7 days after the MI in the aforementioned study [89]. 

One of the frequently reported targets of miR-93 is PTEN, which is mostly verified in cancer studies [113,114]. At a point in the antioxidant defense system, suppression of PTEN could lead to the suppression of FOXO-mediated antioxidant gene expression because PTEN is known to suppress protein kinase B (AKT) activity and inhibit the PI3K/AKT/NFκB pathway by dephosphorylating phosphatidylinositol-3,4,5-triphosphate (PIP3) and phosphatidylinositol-4.5-biphosphate (PIP2) [115]. Since AKT-mediated phosphorylation of FOXO results in the inactivation and nuclear exclusion of FOXO [103], miR-93-mediated suppression of PTEN may result in the suppression of FOXO-dependent expression of antioxidant genes via enhancement of AKT activity. 

#### 4.1.10. miR-134 

The role of miR-134-5p in CVDs has been examined in a mouse MI model, and it was demonstrated that the MI significantly increased miR-134-5p, while silencing of miR-134-5p improved myocardial angiogenesis and apoptosis [116]. Furthermore, it was also reported that miR-134-5p was up-regulated in the blood of acute myocardial infarction (AMI) patients and H/R-injured CMs [117]. In the latter study, it was demonstrated that an X-linked inhibitor of apoptosis protein (XIAP) was the direct target of miR-134-5p, and its down-regulation by miR-134-5p contributed to increased oxidative stress following H/R injury. 

XIAP is known as an endogenous inhibitor of caspases and is an important regulator of cell death [118]. Furthermore, more importantly, it has been reported that XIAP increased mitochondrial antioxidants such as SOD2 through NF-κB activation [119]. Additionally, it was also demonstrated that other antioxidant regulators, such as TRX2, NQO1, and HO-1, were significantly decreased in XIAP knockout cells [120], suggesting the important role of XIAP in regulating cellular oxidative stress. Therefore, currently available evidence strongly suggests that miRNAs targeting XIAP, such as miR-134-5p, will have a negative impact on the cardiovascular system. The observation that down-regulation of miR-134-5p inhibited both the H/R-induced oxidative stress and apoptosis in CMs supports such speculation [117]. 

#### 4.1.11. miR-195

In a mouse model of MI, miR-195 expression was reported to be up-regulated, and the inhibition of miR-195 reduced MI-induced fibrosis and improved cardiac function [121]. In another study that examined the role of miR-195 in H/R-injured rat CMs, it was demonstrated that the H/R injury increased the expression of miR-195, causing cellular apoptosis [122]. In a more comprehensive study where the expression of miR-195 was examined using both human samples and animal models, it was demonstrated that the expression of miR-195 was up-regulated in the myocardial sample of heart failure patients and also in the TAC or MI-induced mouse models of heart failure [123]. In the last study, SIRT3 was identified as a direct target of miR-195, and it may be the link that connects miR-195 to oxidative stress regulation.

SIRT3 is one of the sirtuins located in the mitochondria and an important regulator of oxidative stress that deacetylates a range of substrates involved in both ROS production and detoxification [124]. SIRT3 is known to promote the antioxidant defense system; for example, it deacetylates the lysine 68 of MnSOD to activate this antioxidant enzyme [125] and, thus, reduces mitochondrial oxidative stress [126]. On the other hand, deficiency in SIRT3 has been reported to decrease the activity of MnSOD, increasing susceptibility to MI injury [127]. Another example of SIRT3-mediated activation of the antioxidant defense involves isocitrate dehydrogenase 2 (IDH2). IDH2 is a mitochondrial enzyme regulated by SIRT3, and it converts NADP+ to NADPH and, therefore, promotes GSH production by supplying NADPH to glutathione reductase (GSR) [128]. Consequently, SIRT3-mediated deacetylation of IDH2 increases cellular GSH and renders cellular protection against oxidative stress [129]. 

Furthermore, SIRT3 can also regulate the expression of antioxidant enzymes by enhancing the transcriptional activity of FOXO3a and PGC-1α. By forming a complex with FOXO3a, SIRT3 enhances the DNA binding of FOX3a to the promoters of antioxidant genes such as MnSOD and CAT [130,131]. In the case of PGC-1α, its transcriptional activity is mainly regulated by cAMP Response Element-Binding Protein (CREB) [132], and SIRT3 can initiate the transcriptional activation of PGC-1α by enhancing CREB phosphorylation [133]. Therefore, increased expression of miR-195 under pathologic conditions is expected to have a negative impact on the cardiovascular system in terms of oxidative stress. 

#### 4.1.12. miR-208 

MiR-208 is one of the cardiac-enriched miRNAs that play a crucial role in the cardiovascular system [134]. Its expression has been found to be increased in patients with heart failure [135,136] and in the myocardial tissue of rats with early MI [137]. Furthermore, it has been demonstrated that the suppression of miR-208 reduced cardiac remodeling while improving cardiac function [138]. Regarding its role in the regulation of oxidative stress, it has been reported that the activity of antioxidant enzymes such as CAT and SOD was significantly decreased in patients with CVDs, while the expression of miR-208 was significantly increased [136], suggesting its pro-oxidative stress role. It was difficult to find studies that examined the effect of miR-208 on the generation of ROS or oxidative stress regulation with a clear explanation of the underlying mechanisms. However, one study that investigated the role of miR-208 in H_2_O_2_-induced CM injury may provide a hint regarding the underlying mechanisms [139].

According to this particular study, delivery of exogenous miR-208a aggravated the H_2_O_2_-induced oxidative stress in CMs by targeting activated protein C (APC). APC is a well-known natural anticoagulant protein that inhibits thrombin generation by inactivating factors (F) Va and VIIIa [140]. However, interestingly, it has also been reported that APC plays antioxidant roles [141] and exerts a cardio-protective effect by activating AMP-activated protein kinase (AMPK) signaling [142]. Considering that AMPK signaling can stimulate the Nrf2/HO-1 signaling axis [143] and trans-activate some of the Nrf2 target genes [144], it may not be far-fetched speculation that down-regulation of APC by miR-208 may result in an increase in oxidative stress by down-regulating the AMPK signaling and subsequent activation of Nrf2. Nevertheless, such a suggested mechanism should remain as a speculation until empirically verified. 

#### 4.1.13. miR-217 

MiR-217 is known as the most highly expressed miRNA during EC aging [145], and the up-regulation of miR-217 has been demonstrated in a mouse model of arrhythmogenic cardiomyopathy, as well [146]. Regarding its role in CVDs, its aberrant expression has been linked to the progression of CVDs such as atherosclerosis and cardiac dysfunction [147], cardiac hypertrophy [148], and myocardial I/R injury [149]. Through these studies, some of the direct targets of miR-217 have been identified, and SIRT1 and PTEN are probably the most relevant targets of miR-217 in terms of oxidative stress regulation.

As discussed previously, targeting SIRT1 may increase oxidative stress by decreasing the deacetylation of FOXO3a and the subsequent transcription of antioxidant genes such as MnSOD and CAT (please see Section 4.1.8 miR-34a). Such miR-217-induced suppression of SIRT1 and the resultant increase in oxidative stress has been demonstrated in non-cardiovascular cells as well [150]. In addition, the suppression of PTEN can result in the suppression of FOXO-dependent expression of the antioxidant genes by enhancing the AKT activity (please see Section 4.1.9 miR-93). 

#### 4.1.14. miR-410 

The expression of miR-410 was reported to be significantly increased following I/R injury in a mouse model, and miR-410 directly targeted high-mobility group box 1 (HMGB1), resulting in the suppression of mitophagy [151]. Additionally, it was demonstrated that the expression of miR-410 significantly increased in a mouse model of atherosclerosis [152]. HMGB1 is originally known as a chromatin-binding nuclear protein that facilitates DNA bending, modulation of transcription factor activity, and DNA repair by binding to the minor groove of DNA [153]. As a damage-associated molecular pattern (DAMP) molecule, HMGB1 has been associated with the pathogenesis of various diseases, including CVDs [154], and a recent study demonstrated that HMGB1 mediated the impinging flow-induced oxidative stress in ECs [155]. One of the feasible mechanisms by which HMGB1 increases oxidative stress may involve the modulation of the activity of NADPH oxidase (NOX). 

NOX is a membrane-bound protein that mainly transfers electrons across the plasma membrane to molecular oxygen, thereby generating the superoxide anion and ROS [156]. Regarding the role of HMGB1 in modulating NOX activity, it has been reported that HMGB1 activated NOX by interacting with toll-like receptor 4 (TLR4) or the receptor for advanced glycation end-product (RAGE) and, consequently, increased the ROS production [157,158]. Therefore, at least in terms of oxidative stress, it can be stated that miR-410 has a protective effect on the cardiovascular system. Nevertheless, modulation of miR-410 produced contradicting results in different types of CVD models [152,159,160], suggesting its role in CVDs may vary depending on the specific pathologic conditions. Therefore, its role in CVDs needs to be carefully interpreted in a given context. 

#### 4.1.15. miR-539

In a mouse model of MI, it was demonstrated that the expression of miR-539 significantly increased, and miR-539 directly targeted O-GlcNAcase (OGA), one of the important modulators of O-GlcNAcylation [161]. O-GlcNAcylation refers to a nutrient- and stress-responsive post-translational modification that attaches/removes the O-linked N-acetylglucosamine (O-GlcNAc) moieties to/from the Ser and/or Thr residues in proteins [162]. While O-GlcNAc transferase (OGT) catalyzes the addition of O-GlcNAc, OGA facilitates the removal of O-GlcNAc. Evidence indicates that altered levels of O-GlcNAcylation are linked to many pathological conditions, including CVDs [163,164]. For example, overexpression of OGA in neonatal rat cardiac myocytes (NRCMs) increased cytotoxicity following H/R injury, and it was abrogated by small interfering RNA (siRNA) specific to OGA [165]. Interestingly, the same group that conducted that particular study later demonstrated that the overexpression of OGT decreased the H/R-induced ROS production, while the overexpression of OGA produced the opposite result in NRCMs [166]. Such O-GlcNAcylation-mediated regulation of ROS production may be the result of modulating the O-GlcNAcylation status of the Nrf2/Keap1 pathway.

Nrf2 is an oxidative stress-responsive transcription factor, and its physiologic level is maintained by the cytoplasmic Keap1 adaptor protein and Cul3 E3 ubiquitin ligase, as briefly discussed above (please see Section 4.1.6 miR-27). In addition, studies have indicated that there is a functional connection between the O-GlcNAcylation and the Nrf2 pathway. To be more specific, there is an inverse correlation between global O-GlcNAcylation and Nrf2 level, as well as the subsequent antioxidant response. For example, the down-regulation of OGT resulted in an increase in Nrf2 target gene expression in various in vitro systems [167,168]. Also, α-lipoic acid (LA)-induced decrease in global O-GlcNAcylation increased the nuclear translocation of Nrf2 and the subsequent expression of the antioxidant enzymes such as SOD and CAT in animal models [169,170]. Furthermore, OGA inhibitors decreased the Nrf2 level and the Nrf2 target gene expression altogether in neuroblastoma cells [171], suggesting down-regulation of OGA can lead to the down-regulation of the Nrf2 target antioxidant gene expression. Therefore, OGA-targeting miR-539 may have a negative impact on the cardiovascular system by suppressing the expression of Nrf2-dependant antioxidant genes.

#### 4.1.16. miR-696

In a previous study that examined the differential expression of mitochondria-associated miRNAs in a TAC-induced heart failure model of animals, the expression of miR-696 was significantly increased following TAC surgery [172]. One of the reported biological functions of miR-696 is to regulate fatty acid oxidation by targeting PGC-1α [173]. As a transcriptional coactivator, PGC-1α has been implicated in cardiac mitochondrial biogenesis and cardiac mitochondrial energy production [174,175], suggesting it is a critical regulatory molecule in the control of cardiac mitochondrial number and function. In addition, PGC-1α has been associated with a number of inflammatory and metabolic diseases as a key regulator of oxidative stress and metabolic pathways [176,177]. As previously discussed (please see Section 4.1.4 miR-22), down-regulation of PGC-1α can decrease fatty acid oxidation and mitochondrial biogenesis, while it can increase ROS production in the heart [79]. This inverse correlation between PGC-1α and ROS production may be achieved by the PGC-1α-mediated expression of the mitochondrial antioxidant proteins [80]. Consequently, it can be speculated that the increased expression of miR-696 may suppress the PGC-1α expression and thereby increase ROS production. 

The miRNAs discussed in this section are listed in the following Table 1. 

### 4.2. Down-Regulated miRNAs in CVDs and Their Possible Roles in the Oxidative Stress Regulation

#### 4.2.1. miR-129

Previous studies have reported that the expression of miR-129 was down-regulated in various experimental models of CVDs, such as CHF [178], myocardial I/R injury [179,180,181], and Ang II-induced CM hypertrophy [182]. More recently, the down-regulation of miR-129 in the cardiac tissue of patients with heart failure has also been reported [183]. These studies also have validated some of the targets of miR-129, and among them, Keap1 [182] and HMGB1 [180] could be meaningful targets in terms of oxidative stress regulation. 

Keap1 is a negative regulator of Nrf2 that facilitates the expression of the antioxidant enzymes such as GST, SOD, HO-1, and NQO1 [93]. Under oxidative stress, the stress-sensing cysteine residue of Keap1 is modified so that it can no longer ubiquitinate the Nrf2 for degradation by Cul3 [94,95]. This can lead to the accumulation and nuclear translocation of Nrf2, consequently inducing the transcription of Nrf2-dependent antioxidant genes [96] (please see Section 4.1.6 miR-27). Therefore, down-regulation of Keap1 by miR-129 would result in the enhancement of the transcriptional activity of the Nrf2, evoking the expression of the antioxidant genes. 

As for the case of HMGB1, it may increase oxidative stress by modulating the activity of NOX, as previously discussed (please see Section 4.1.13 miR-410). Briefly, HMGB1 can activate NOX by interacting with TLR4 or RAGE [157,158]. As a superoxide anion-generating enzyme [156], the activated NOX will contribute to an increase in oxidative stress. Thus, down-regulation of HMGB1 by miR-129 may have a protective effect on the cardiovascular system in terms of oxidative stress regulation. 

#### 4.2.2. miR-130

It has been reported that the expression of miR-130 was down-regulated in cFBs exposed to hypoxic conditions and in an animal model of MI [184], as well as in an animal model of I/R injury [185]. In the latter study, HMGB2 was identified as a direct target of miR-130, and it may tell something from which the role of miR-130 in the regulation of oxidative stress can be inferred. HMGB2 is one of the HMGB domain proteins, and it shares 80% of amino acid sequences with HMGB1, the most common and well-studied HMGB domain protein [186]. Furthermore, just as in the case of HMGB1, HMGB2 can interact with RAGE to initiate downstream signaling [187]. In fact, it has been reported that HMGB2 down-regulated the Nrf2/HO-1 signaling pathway by interacting with RAGE, resulting in the decreased expression of antioxidant proteins [188]. Therefore, maintaining a physiologic level of miR-130 can be cardio-protective in terms of oxidative stress. The observation that the overexpression of miR-130a-5p alleviated myocardial I/R injury-induced oxidative stress in mice [185] supports such speculation. 

#### 4.2.3. miR-133

The expression of miR-133 has been reported to be down-regulated in human MI patients [189,190]. Furthermore, in an in vitro CM model, hypoxia significantly decreased the expression of miR-133, while the overexpression of miR-133 prevented hypoxia-induced CM apoptosis [191]. Regarding the role of miR-133 in the regulation of oxidative stress, it was difficult to find studies that examined the impact of miR-133 on oxidative stress in the cardiovascular system or cardiovascular-related cells altogether. However, although it was investigated in a non-cardiovascular system, there was a study whose findings might shed some light on the possible role of miR-133 in the regulation of oxidative stress [192]. 

According to that particular study, miR-133 attenuated H_2_O_2_-induced oxidative stress by directly targeting BTB and CNC Homology 1 (BACH1). BACH1 belongs to the Cap‘n’Collar type of basic region leucine zipper transcription factor family, and it binds to Maf recognition elements (MAREs) in a form of complex with small Maf (sMaf) proteins, suppressing corresponding genes [193]. However, when there is oxidative stress, BACH1 translocates out of the nucleus, while the Nrf2 dissociated from Keap1 translocates into the nucleus to bind to MAREs, thus activating oxidative stress response genes [194]. In other words, BACH1 can act as a transcriptional repressor of Nrf2 that inhibits the interaction between the Nrf2 and its corresponding ARE in the nucleus. Therefore, down-regulation of miR-133 can result in an increase in BACH1, and in turn, it can suppress Nrf2-dependant antioxidant gene expressions. 

#### 4.2.4. miR-142

The expression of miR-142 has been reported to be down-regulated in a rat model of abdominal aortic constriction (AAC)-induced cardiac hypertrophy [195] and also in a pig model of coronary microembolization (CME)-induced MI [196]. In addition, H/R injury significantly down-regulated the expression of miR-142 in mouse CMs, and the augmentation of miR-142 suppressed apoptosis and fibrosis of CMs by targeting HMGB1 [197]. 

As discussed earlier (please see Section 4.1.13 miR-410), HMGB1 can increase oxidative stress by activating NOX, a membrane-bound electron transfer protein that generates the superoxide anion and ROS [156]. Additionally, although it was not demonstrated in the cardiovascular system, miR-142 can directly target Nrf2 [198]. Therefore, it is theoretically feasible that miR-142 can contribute to an increase in oxidative stress by dysregulating the Nrf2/ARE signaling pathway. 

#### 4.2.5. miR-148

Previous studies have reported that the expression of miR-148 was down-regulated in myocardial I/R-injured rats [199] and in human atherosclerosis plaque [200]. Among the experimentally validated targets of miR-148, pyruvate dehydrogenase kinase (PDK4) [199] and sestrin2 (SESN2) [201] may be worth discussing for the role of miR-148 in the regulation of oxidative stress. 

PKD is an enzyme located in the outer mitochondrial membrane, and it can negatively regulate the activity of pyruvate dehydrogenase (PDH), another enzyme located in the outer mitochondrial membrane, by phosphorylating one of the PDH’s subunits [202]. Furthermore, PDH is the E1α subunit of pyruvate dehydrogenase complex (PDC) that converts pyruvate into acetyl-CoA by a process called pyruvate decarboxylation. The activity of PDC is regulated via reversible phosphorylation of three serine residues on PDH. Therefore, phosphorylation of PDH by the PDK can decrease the PDC activity [203]. According to a previous study that investigated the impact of PDC deficiency on cellular ROS production, PDC deficiency resulted in the accumulation of superoxide anion, decreased MnSOD activity, and down-regulation of uncoupling protein 2 (UCP2) expression [204]. Considering MnSOD is a superoxide detoxifying enzyme [205] and UCP2 protects cells from oxidant damage by scavenging superoxide anion [206], deficiency of PDC can lead to cellular superoxide anion accumulation by impairing the mitochondrial antioxidant defenses, such as MnSOD and UCP2. 

Therefore, it may be possible that enhanced PDK4 activity due to the down-regulation of miR-148 increases oxidative stress by increasing PDH phosphorylation while decreasing the activity of PDC. A recent study that demonstrated that the inhibition of PDK4 suppressed oxidative stress and inflammation in renal I/R injury may also support such speculation [207].

SESN2 is a member of the highly conserved sestrin gene family and is a stress-responsive gene [208]. SESN2 can alleviate oxidative stress by activating Nrf2 signaling and, thus, counteracting ROS production [209]. Therefore, down-regulation of miR-148 may help to maintain a relatively higher level of SESN2 under cellular stress. In this context, down-regulation of miR-148 may be beneficial. Nevertheless, whether the down-regulation of miR-148 exerts a detrimental effect via targeting PDK4 or a protective effect via targeting SESN2, or neither, should be empirically validated. 

#### 4.2.6. miR-199

According to a previous study that examined the expression of miRNAs in the myocardial tissue of patients who went through coronary artery bypass graft (CABG) surgery, the expression of miR-199 was decreased approximately 2-fold compared to healthy controls [210]. Known targets of miR-199 include, but are not limited to, hypoxia-inducible factor 1 alpha (Hif-1α) [211], SIRT1 [211], and Brahma-related gene 1 (BRG1) [212]. Among them, SIRT1 and Brg1 may be the most relevant targets of miR-199 in regard to oxidative stress regulation. 

As briefly discussed in Section 4.1.7 miR-34a, SIRT1 can exert an antioxidant response by modulating FOXO3a, which regulates the expression of genes involved in the antioxidant defense system [103]. The transcriptional activity of FOXO3a is known to be dependent on its post-translational modifications, such as deacetylation or phosphorylation [106], and SIRT1 is known to deacetylate FOXO3a inducing antioxidant responses [107]. Therefore, down-regulation of miR-199 may help to maintain a certain level of SIRT1 so that it can facilitate the antioxidant response.

BRG1 is the core ATPase subunit of a large chromatin-remodeling complex and regulates the transcription process by altering the chromatin structure [213]. Regarding its impact on the regulation of oxidative stress, it has been demonstrated that the overexpression of BRG1 increased the expression of Nrf2 with increased antioxidant activity in hepatocytes [214]. In another study, BRG1 overexpression alleviated hepatic I/R injury by up-regulating a range of antioxidant enzymes, including NQO1, SOD, glutamate-cysteine ligase catalytic subunit (GCLC), and glutathione S-transferase alpha 1 (GSTα1) [215]. Furthermore, a more recent study reported that BRG1 played an antioxidant role in MI as well by up-regulating Nrf2 expression and the subsequent HO-1 expression [216]. 

#### 4.2.7. miR-204

Mir-204 is one of the miRNAs down-regulated in human MI [217] and PAH [218], and its down-regulation has also been reported in a rat model of myocardial I/R injury [219]. Additionally, the involvement of miR-204 in the regulation of oxidative stress has been demonstrated in a study that examined the role of miR-204 in endoplasmic reticulum (ER) stress-induced endothelial dysfunction [220]. According to this particular study, miR-204 promoted vascular ER stress and endothelial dysfunction by targeting SIRT1. Furthermore, ER stress is known to increase ROS production, leading to oxidative stress [221], and miR-204 was found to be indispensable in translating ER stress into ROS production in that study. Although the authors did not investigate whether the miR-204-induced ROS production was due to the down-regulation of the SIRT1-dependant antioxidant genes, considering the anti-oxidative role of SIRT1 (please see Section 4.1.4 and Section 4.1.8), such possibility cannot be simply excluded without experimental validation. 

#### 4.2.8. miR-381

It has been reported that the expression of miR-381 was down-regulated in human atherosclerosis plaque [222] and in the serum of coronary heart disease patients [223]. Additionally, high glucose treatment to simulate hyperglycemic conditions also significantly decreased the expression of miR-381 in VSMCs [224]. Through these studies, HMGB1 [187] and cyclooxygenase 2 (COX-2) [222] have been identified as direct targets of miR-381, and miR-381 may participate in the regulation of oxidative stress by modulating them. 

A possible oxidative stress-related role of HMGB1 is to activate NOX. NOX facilitates electron transfer across the plasma membrane to molecular oxygen, and as a result, superoxide anion and ROS are generated [156]. HMGB1 can activate NOX in a TLR4 or RAGE-dependent manner, thereby increasing ROS production [157,158].

In the case of COX-2, it can modulate the transcriptional activity of Nrf2 by facilitating the formation of electrophilic fatty acid oxo-derivatives (EFOXs). COX-2 can oxygenate a variety of fatty acids [225] so that it can convert ω-3 fatty acids docosahexaenoic acid (DHA) and eicosapentaenoic acid (EPA) into EFOX-D6 and EFOX-D5, respectively [226]. EFOXs are also reactive electrophilic species (RES) that have an electron-withdrawing functional group. The electron-withdrawing functional group of EFOXs promotes addition reactions with cellular nucleophiles such as cysteine and histidine residues of protein [227]. For example, when such addition occurs in the critical cysteine residues of Keap1, it induces a dissociation of the Keap1-Cul3 ubiquitination system, leading to the accumulation of free Nrf2 that can translocate into the nucleus and initiate the expression of antioxidant genes [228].

As such, the targets of miR-381 include both pro-oxidative and anti-oxidative molecules. Therefore, it is difficult and imprudent to decisively state whether miR-381 is cardio-protective or not. Since CVDs are multifactorial diseases, the overall impact of miR-381 on the cardiovascular system has to be carefully evaluated and interpreted in a given context.

#### 4.2.9. miR-708

Down-regulation of miR-708 in CVDs has been reported previously. One study reported that the expression of miR-708 was down-regulated in CMs exposed to hypoxia, MI-injured rat hearts, and the serum samples of the MI patients [229]. In another study, myocardial I/R injury down-regulated the expression of miR-708, and HMGB1 was identified as one of the direct targets of miR-708 [230]. 

As briefly discussed previously (please see Section 4.1.13 miR-410), HMGB1 may participate in the regulation of oxidative stress by modulating the activity of NOX. In fact, miR-708 showed cardioprotective effects, such as decreased ROS production and increased SOD expression in the I/R injury study above, and restoring HMGB1 expression abrogated the cardioprotective effect of miR-708 [230]. 

Additionally, it has been reported that miR-708 prevented ROS-induced apoptosis in osteoblasts by targeting PTEN [231]. Considering the suppression of PTEN could lead to the suppression of the FOXO-mediated antioxidant gene expression by inhibiting the AKT-mediated phosphorylation of FOXO [103], the observed anti-apoptotic effect of miR-708 may partially be owed to the miR-708-mediated antioxidant effect.

The miRNAs discussed in this section are listed in the following Table 2.

### 4.3. MiRNAs Whose Expression Varied in CVDs and Their Possible Roles in the Oxidative Stress Regulation

#### 4.3.1. miR-1

MiR-1 is one of the abundant miRNAs of the heart [232], and its close association with IR injury has been reported [233]. The expression of miR-1 in CVDs is found to be highly varied, even in similar experimental settings. For example, the expression of miR-1 was reported to be down-regulated in human MI [189,234]. On the other hand, it was demonstrated that miR-1 was up-regulated following MI induced by coronary artery ligation in mice [235]. This perplexing discrepancy is also observed in the case of myocardial I/R injury models using rodents. In a rat model of I/R injury, the expression of miR-1 was found to be decreased following the I/R injury [236], while in a mouse model of I/R injury, miR-1 was up-regulated following the I/R injury [237]. 

These somewhat contradicting results tell us that there may be a species-dependent difference in regulating the expression of certain miRNAs, or the expression of certain miRNAs is so meticulously regulated that subtle differences in experimental settings can lead to quite opposite outcomes. Supporting such speculation, it has been demonstrated that a low concentration (30 μM) of H_2_O_2_ decreased miR-1 expression, while a high concentration (200 μM) of H_2_O_2_ increased miR-1 expression [238]. Regardless of its varying expression in CVDs, it is clear that miR-1 plays an important role in regulating cellular oxidative stress since its direct target includes a number of antioxidant genes, such as SOD1, GCLC, and G6PD [239]. 

SOD1 is an important scavenger protein that converts the superoxide radical into molecular oxygen and hydrogen peroxide. Although superoxide itself is not a strong oxidizing agent, it can be potentially deleterious if its enzymatic removal by SOD-catalyzed dismutation reaction is insufficient [240]. Its cardio-protective role has also been demonstrated in an animal model of I/R injury [241], suggesting miRNA-mediated down-regulation of SOD1 may have a negative impact on the cardiovascular system. 

The reduced form of GSH, as opposed to the oxidized glutathione disulfide (GSSG), can scavenge a wide variety of reactive species such as superoxide anion, hydroxyl radical, and singlet oxygen by donating electrons and becoming oxidized to form GSSG. Furthermore, it can also function as a cofactor for antioxidant and detoxification enzymes such as GPx, GST, and glyoxalases (GLO) [242,243]. The first rate-limiting step of GSH synthesis is the generation of gamma-glutamyl cysteine from L-glutamate and cysteine by glutamate-cysteine ligase (GCL). GCL is a heterodimeric enzyme composed of a catalytic (GCLC) and modulatory (GCLM) subunit, and its enzymatic activity is solely dependent on the GCLC subunit [244]. Considering that the increase in GCLC up-regulated the GSH production in CMs, protecting them from oxidative injury [245], miR-1-mediated down-regulation of GCLC can lead to the down-regulation of GSH and the subsequent increase in oxidative stress. 

Another important pathway for the production of GSH and NHDPH is the pentose phosphate pathway (PPP), and G6PD is the rate-limiting enzyme of the PPP [246]. NADPH is an important free radical deactivator, and G6PD is responsible for maintaining adequate levels of NADPH inside the cell [247]. It has been reported that the activity of G6PD is promptly increased in CMs exposed to oxidative stress [248], and thus, miR-1-mediated down-regulation of G6PD would have a negative impact on the cardiovascular system, especially in terms of the oxidative stress regulation. 

#### 4.3.2. miR-103

The reported expression pattern of miR-23 in CVDs is rather inconsistent. Studies have reported that the expression of miR-103 was down-regulated in various experimental CVD models, such as pressure overload-induced cardiac hypertrophy [249] and H_2_O_2_-treated ECs [250,251]. On the other hand, the expression of miR-103 was found to be up-regulated in isoprenaline-induced MI in mice [252]. Among many different possible targets of miR-23, Bcl-2/adenovirus E1B 19 kDa interacting protein (BNIP3) seems to be worth looking into for this review. 

Both of the aforementioned studies that examined the role of miR-103 in H_2_O_2_-stimulated ECs identified BNIP3 as a direct target of miR-103. Also, it was demonstrated that the augmentation of miR-103 suppressed BNIP3 expression and H_2_O_2_-induced ROS production altogether [250,251]. Furthermore, according to a study that examined the role of BNIP3 in the pathogenesis of heart failure, the overexpression of BNIP3 increased ROS production while decreasing the mitochondria membrane potential in rat CMs [253], strongly suggesting miR-103-mediated regulation of oxidative stress by targeting BNIP3.

Although it is not directly linked to the production or removal of ROS, BNIP3 may function as a kind of antioxidant defense by inducing autophagy. Autophagy refers to a cellular degradation and recycling process for damaged organelles and macro-molecules [254]. Especially, the removal or degradation of the mitochondria by autophagy is called mitophagy [255]. Under pathologic conditions, mitochondria damage and/or dysfunction can occur, and such damaged or dysfunctional mitochondria themselves serve as a significant source of oxidative stress [256]. In that sense, mitophagy can be considered a component of the cellular antioxidant defense system that prevents pathologic mitochondrial ROS generation [257]. Since BNIP3 is known to be a strong inducer of mitophagy under pathologic conditions [258], miRNA-mediated alteration of BNIP3 expression can either promote or disrupt the process of mitophagy, thereby affecting ROS production. 

#### 4.3.3. miR-132

Altered expression of miR-132 in many different types of CVD models has been reported, and it varies depending on the type of pathologic stimuli. For example, its expression was found to be up-regulated in a mouse model of I/R injury [259] and also in a mouse model of hindlimb ischemia [260]. Furthermore, its up-regulation has been reported in mice with TAC-induced cardiac hypertrophy [261,262]. On the other hand, the expression of miR-132 has been reported to be down-regulated in rodent models of MI, both in rats [263,264] and mice [265], indicating its expression may vary depending on the type of the pathologic stimuli.

As the oxidative stress regulation-related targets of miR-132, there are FOXO3a, SIRT1, and PTEN [266]. As discussed previously (please see Section 4.1.8 miR-34a), FOXO3a is a well-known transcription factor of antioxidant genes such as MnSOD and CAT, both of which have FOXO3a binding sites in their promoters [103,104]. Furthermore, SIRT1 can enhance the transcriptional activity of FOXO3a by deacetylating the FOXO3a and, therefore, inducing antioxidant responses [107]. In addition, suppression of PTEN can result in the suppression of FOXO-dependent expression of antioxidant genes via enhancement of AKT activity (please see Section 4.1.9 miR-93). 

#### 4.3.4. miR-206

According to a previous study, MI and H/R down-regulated the expression of miR-206 in rats and in rat cardiomyocytes, respectively [267]. However, another study that examined the expression of miR-206 in a mouse model of MI reported that the expression of miR-206 was up-regulated [268], and such MI-induced up-regulation of miR-206 was also demonstrated in yet another study that used a rat model of MI [269].

Regarding its possible role in the regulation of oxidative stress, it was difficult to find studies where the role of miR-206 was clearly demonstrated with empirically validated targets in the cardiovascular system. Nevertheless, a study examining the role of miR-206 in microparticle-induced asthma may provide useful information for projecting its impact on the cardiovascular system [270]. In this particular study, SOD1 was empirically validated as a direct target of miR-206, and miR-206-mediated suppression of SOD1 eventually resulted in the accumulation of ROS. Considering SOD1 is an important scavenger protein that converts the superoxide radical into molecular oxygen and hydrogen peroxide, miR-206-mediated down-regulation of SOD1 is expected to have a negative impact on the cardiovascular system (please see Section 4.3.1 miR-1). 

#### 4.3.5. miR-214

The expression of miR-241 has been examined in various types of CVD models, and the most frequently used model is MI in rodents. The expression of miR-241 was found to be up-regulated in mouse models of MI [271,272] and also in a rat model of MI [273]. Also, myocardial I/R injury [274] and AAC-induced cardiac hypertrophy [275] increased the expression of miR-214 in mice and rats, respectively. Beta-adrenergic receptors (β-ARs) are the dominant adrenergic receptors in the heart, and they are known to play an important role in cardiac fibrosis [276]. Thus, β-AR agonist isoproterenol (ISO) has long been used for cardiac fibrosis models [277], and IOS treatment increased the expression of miR-214 in rats [278,279]. On the other hand, when AngII-infusion was used to induce cardiac fibrosis in mice, the expression of miR-214 was found to be down-regulated [280]. 

Among validated targets of miR-214, mitochondrial NAD(P)+-dependent malic enzyme (ME2) and GSR may facilitate miR-214-mediated regulation of oxidative stress. For ME2, it has been reported that miR-214 down-regulated ME2 expression in a mouse model of MI, causing an increase in ferroptosis, a unique iron-dependent cell death [271]. Malic enzymes, such as ME3, mediate the oxidative decarboxylation of L-malate that produces CO_2_ and pyruvate, and in the process, NADP+ is reduced to NADPH, an important ROS-reducing antioxidant [281]. Regarding its role in the regulation of cellular ROS, it has been demonstrated that silencing ME2 results in a decrease in NADPH and a subsequent increase in ROS [282].

As for the GSR, it has been demonstrated that miR-214 exacerbated alcohol-induced oxidative stress by directly targeting GSR in liver cells [283]. GSR is one of the antioxidant enzymes that reduces GSSG to GSH, an important cellular antioxidant (please see Section 4.3.1 miR-1). For example, as cellular ROS, such as H_2_O_2_, increases, GSH can turn it into water with the help of GPx. In this process, GSH is converted to its oxidized form, GSSG. It is the GSR that facilitates the conversion of GSSG into GSH in the presence of NADPH to further detoxify the cellular ROS [284]. Therefore, it is feasible that miR-214 participates in the regulation of oxidative stress by modulating the expression of these antioxidant defense system-related targets. 

The miRNAs discussed in this section are listed in the following Table 3.

## 5. Concluding Remarks

For decades, both ROS and miRNAs have been extensively studied for their regulatory roles in the development and progression of CVDs, providing detailed descriptions of specific interactions between them that could be used in the development of diagnostic and therapeutic strategies for CVDs. However, due to the complexity of the interaction between those two pleiotropic biological entities, there still remains a lot more to be investigated than already has been. For more efficient and productive future investigation, this review tried to explain how to develop research topics from seemingly distant and scattered information on the subject and also to provide examples generated by following the steps shown in Figure 1. The flow chart may be stating the obvious, but getting back to basics never hurts. It will definitely help to generate a scientifically sound and reasonable hypothesis worthy of testing. However, it should be noted that the scenarios provided in this review must be treated as mere speculations until proven otherwise since they have not been empirically validated or confirmed. Similarly, the expression pattern of certain miRNAs described in this review should not be taken as something absolute because the expression of miRNA can be easily changed by even a subtle change of external stimuli. Again, this narrative review aims to provide mechanistic insights on possible oxidative stress regulating roles of miRNAs in CVDs, and if any of those scenarios provided in this review helps anyone to formulate his/her own scenarios or provide a clue to interpret perplexing results, that will fulfill the purpose of this review more than enough. 

## Figures and Tables

**Table 1:** MiRNAs reported to be UP-regulated in CVDs with their validated targets expected to be involved in the regulation of oxidative stress.

miRNA	Altered Expression Reported in	Empirically Validated Oxidative Stress Regulation-Related Target	Probable Oxidative Stress Regulation- Related Outcome
miR-15	^1^ I/R injury in porcine and mice [48]	^2^ SIRT4 [50]	Increased SIRT3 binding to ^3^ SOD2, enhancing SOD2 activity [52]
	Dicer depletion-induced cardiac dysfunction [49]		Increased fatty acid oxidation and oxygen consumption [53]
miR-17-92 cluster	Hypoxia-induced ^4^ PAH in rats [56]	^5^ HO-1 [59]	Decreased HO-1-mediated ^6^ ROS scavenging [60]
	Human ^7^ HCM [58]	^8^ PTEN [57]	Suppressed ^9^ FOXO-mediated antioxidant gene expression [115]
miR-21	Human ^10^ ARVC [62]	^11^ KRIT1 & SOD2 [70]	Increased ROS and decreased SOD2 [71]
	Human HCM and ^12^ DCM [63]	SOD3 and ^13^ TNFα [72]	Increased ROS and suppressed SOD2 expression [73]
miR-22	^14^ TAC- or isoproterenol-induced cardiac hypertrophy in mice [75,76].	SIRT1 [78]	Unusually, miR-22 increases SIRT1 expression [83]; thus, it can lead to transcriptional activation of ^15^ PGC-1α [82]
	In vitro phenylephrine or ^16^ AngII treatment [75,77]	PGC-1α [78]	Increased ROS production [79] Decreased PGC-1α-mediated expressions of antioxidant genes [80]
miR-23	^17^ MI in mice [85]	^18^ PPARα [72]	Down-regulation of PPARα-mediated expression of antioxidant genes such as SOD1, 2, ^19^ CAT, and HO-1 [89,90]
	I/R injury in rats [86]		
miR-27	TAC-induced hypertrophy in mice [91] MI-induced ^23^ HF in rats [92]	^20^ Nrf2 [92]	Suppressed Nrf2-mediated expression of antioxidant genes such as ^21^ GST, SODs, HO-1, and ^22^ NQO1 [93]
miR-28	MI-induced HF in rats [92]	Nrf2 [97]	Suppressed Nrf2-mediated expression of antioxidant genes [93]
miR-34a	MI in rats [98,99]	SIRT1 [102]	Decreased SIRT1-mediated deacetylation of FOXO3a, thus suppressing antioxidant responses [107]
miR-93	MI in mice [109]	PTEN [112]	Suppressed FOXO-mediated antioxidant gene expression [115]
	Carotid artery balloon injury in rats [110] Hindlimb ischemia in mice [111]		
miR-134	MI in mice [116]	^26^ XIAP [117]	Down-regulation of SOD2 via suppression of XIAP/^27^ NF-κB pathway [119]
	^24^ H/R-injured ^25^ CMs [117]		
miR-195	MI in mice [121]	SIRT3 [123]	Reduced ^28^ MnSOD activation [125] Decreased ^29^ IDH2-mediated conversion of NADP+ to ^30^ NADPH [128] Decreased transcriptional activity of FOXO3a [130,131] and PGC-1α [133]
	H/R-injured CMs [122] Human HF, TAC, or MI-induced HF in mice [123]		
miR-208	Human HF [135]	^31^ APC [139]	Increased oxidative stress via down-regulation of ^32^ AMPK signaling and subsequent down-regulation of Nrf2 activity [142,143,144]
	MI in rats [137] H_2_O_2_ treated CMs [139]		
miR-217	Arrhythmogenic cardiomyopathy in mice [146] Atherosclerosis in mice [147] TAC-induced cardiac hypertrophy in mice [148] I/R injury in mice [149]	SIRT1 [145] PTEN [148]	Decreased SIRT1-mediated deacetylation of FOXO3a, thus suppressing antioxidant responses [107] Suppressed FOXO-mediated antioxidant gene expression [115]
miR-410	I/R injury in mice [151]	^33^ HMGB1 [151]	Down-regulation of HMGB1-mediated ^34^ NOX activation and subsequent ROS production [157,158]
	Atherosclerosis in mice [152]		
miR-539	MI in mice [161]	^35^ OGA [161]	Down-regulation of Nrf2 target antioxidant gene expression [171]
miR-696	TAC-induced HF in mice [172]	PGC-1α [173]	Increased ROS production [79] Decreased PGC-1α-mediated expressions of antioxidant genes [80]

^1^ Ischemia–reperfusion, ^2^ Sirtuin 4, ^3^ Superoxide dismutase, ^4^ Pulmonary artery hypertension, ^5^ Heme oxygenase 1, ^6^ Reactive oxygen species, ^7^ Hypertrophic cardiomyopathy, ^8^ Phosphatase and tensin homolog deleted on chromosome 10, ^9^ Forkhead Box O, ^10^ Arrhythmogenic right ventricular cardiomyopathy, ^11^ Krev/Rap1 interaction trapped-1, ^12^ Dialated cardiomyopathy, ^13^ Tumor necrosis factor alpha, ^14^ Transverse aortic constriction, ^15^ Peroxisome proliferator activated receptor-gamma coactivator 1 alpha, ^16^ Angiotensin II, ^17^ Myocardial infarction, ^18^ Peroxisome proliferator-activated receptor alpha, ^19^ Catalase, ^20^ Nuclear factor erythroid 2–related factor 2, ^21^ Glutathione S-transferase, ^22^ NAD(P)H: quinine oxidoreductase-1, ^23^ Heart failure, ^24^ Hypoxia-reoxygenation, ^25^ Cardiomyocytes, ^26^ X-linked inhibitor of apoptosis protein, ^27^ Nuclear factor kappa-light-chain-enhancer of activated B cells, ^28^ Manganese superoxide dismutase, ^29^ Isocitrate dehydrogenase 2, ^30^ Nicotinamide adenine dinucleotide phosphate hydrogen, ^31^ Activated protein C, ^32^ AMP-activated protein kinase, ^33^ High-mobility group box 1, ^34^ NADPH oxidase, ^35^ O-GlcNAcase.

**Table 2:** MiRNAs reported to be DOWN-regulated in CVDs with their validated targets expected to be involved in the regulation of oxidative stress.

miRNA	Altered Expression Reported in	Empirically Validated Oxidative Stress Regulation-Related Target	Probable Oxidative Stress Regulation- Related Outcome
miR-129	Human HF [183] ^1^ CHF in rats [178]	^2^ Keap1 [182]	Suppressed transcription of Nrf2-dependent antioxidant genes [96]
	I/R injury in rodents [179,180,181] AngII-induced CM hypertrophy [182]	HMGB1 [180]	Enhanced HMGB1-mediated NOX activation and subsequent ROS production [157,158]
miR-130	Hypoxia exposed ^3^ FBs and MI in mice [184] I/R injury in mice [185]	HMGB2 [185]	Suppressed Nrf2/HO-1 signaling pathway, thus decreasing antioxidant protein expression [188]
miR-133	Human MI [189,190]	^4^ BACH1 [192]	Suppressed Nrf2-dependant antioxidant gene expressions [194]
	Hypoxia-exposed CMs [191]		
miR-142	^5^ AAC-induced cardiac hypertrophy in rats [195]	HMGB1 [197]	Enhanced HMGB1-mediated NOX activation and subsequent ROS production [157,158]
	^6^ CME-induced MI in pigs [196] H/R injured CMs [197]		
miR-148	Human atherosclerosis [200] I/R injury in rats [199]	^7^ PDK4 [199]	Increased oxidative stress by increasing ^8^ PDH phosphorylation while decreasing the activity of ^9^ PDC [204,207]
		^10^ SESN2 [201]	Alleviate oxidative stress by activating Nrf2 signaling and, thus, counteracting ROS production [209]
miR-199	^11^ CABG surgery patients [210]	SIRT1 [211] ^12^ BRG1 [212]	Enhanced antioxidant response via deacetylation of FOXO3a [107] Enhanced Nrf2 expression and subsequent HO-1 expression [216]
miR-204	Human MI [217] Human ^13^ PAH [218] I/R injury in rats [219]	SIRT1 [220]	Enhanced antioxidant response via deacetylation of FOXO3a [107]
miR-381	Human atherosclerosis [222] ^14^ HG treated ^15^ VSMCs [224]	HMGB1 [224]	Enhanced HMGB1-mediated NOX activation and subsequent ROS production [157,158]
		^16^ COX-2 [222]	Enhanced Nrf2 transcriptional activity by producing ^17^ EFOXs that can induce Keap1-Cul3 ubiquitination system [226,228]
miR-708	Hypoxia exposed CMs and MI in rats [229] I/R injury in rats [230]	HMGB1 [230] PTEN [231]	Enhanced HMGB1-mediated NOX activation and subsequent ROS production [157,158]
			Enhanced FOXO-mediated antioxidant gene expression [103]

^1^ Chronic heart failure, ^2^ Kelch-like ECH-associated protein 1, ^3^ Fibroblasts, ^4^ BTB and CNC Homology 1, ^5^ Abdominal aortic constriction, ^6^ Coronary microembolization, ^7^ Pyruvate dehydrogenase kinase, ^8^ Pyruvate dehydrogenase, ^9^ Pyruvate dehydrogenase complex, ^10^ Sestrin 2, ^11^ Coronary artery bypass graft, ^12^ Brahma-related gene 1, ^13^ Pulmonary artery hypertension, ^14^ High glucose, ^15^ Vascular smooth muscle cells, ^16^ Cyclooxygenase 2, ^17^ Electrophilic fatty acid oxo-derivatives.

**Table 3:** MiRNAs whose expression varied in CVDs with their validated targets expected to be involved in the regulation of oxidative stress.

miRNA	Altered Expression Reported in	Empirically Validated Oxidative Stress Regulation-Related Target	Probable Oxidative Stress Regulation- Related Outcome IF UP-REGULATED
miR-1	**Up-regulated** MI in mice [235] I/R injury in mice [237]	SOD1 [239] ^1^ GCLC [239] ^4^ G6PD [239]	Decreased enzymatic removal of superoxide by SOD [240] Decreased ^2^ GCL-mediated synthesis of ^3^ GSH [244,245] Down-regulation of ^5^ PPP that produces GSH and NADPH [246,247]
	**Down-regulated** Human MI [189,234] I/R injury in rats [236]		
miR-103	**Up-regulated** Isoprenaline induced MI in mice [252]	^6^ BNIP3 [250,251]	Down-regulated mitophagy, increased mitochondrial ROS generation [256,257,258]
	**Down-regulated** Pressure overload-induced cardiac hypertrophy in rats [249] H_2_O_2_-treated endothelial cells [250,251]		
miR-132	**Up-regulated** I/R injury in mice [259] Hindlimb ischemia in mice [260] TAC-induced cardiac hypertrophy in mice [261,262]	FOXO3a [266] SIRT1 [266] PTEN [266]	Down-regulated FOXO3a-mediated expression of antioxidant genes such as MnSOD and CAT [103,104] Suppressed deacetylation of FOXO3a, down-regulating antioxidant response [107] Suppressed FOXO-mediated antioxidant gene expression [115]
	**Down-regulated** MI in rats [263,264] and mice [265]		
miR-206	**Up-regulated** MI in mice [268] and rats [269]	SOD1 [270]	Decreased enzymatic removal of superoxide by SOD [240]
	**Down-regulated** MI in rats [267] H/R injured CMs [267]		
miR-214	**Up-regulated** MI in mice [271,272] and rats [273] I/R injury in mice [274] AAC-induced cardiac hypertrophy in rats [275] ^7^ IOS induced cardiac fibrosis in rats [278,279]	^7^ ME2 [271]	Decreased NADPH, increased ROS [282]
	**Down-regulated** AngII-induced cardiac fibrosis in mice [280]	^8^ GSR [283]	Decreased GSR-mediated conversion of GSSG into GSH [284]

^1^ Glutamate cysteine ligase catalytic, ^2^ Glutamate-cystein ligase, ^3^ Glutathion, ^4^ Glucose-6-phosphate dehydrogenase, ^5^ Pentose phosphate pathway, ^6^ Bcl-2/adenovirus E1B 19 kDa interacting protein 3, ^7^ Mitochondrial NAD(P)+-dependent malic enzyme, ^8^ Glutathion reductase.
